# Temperate bacteriophage induced in *Pseudomonas aeruginosa* biofilms can modulate bacteriophage and antibiotic resistance

**DOI:** 10.1016/j.bioflm.2025.100333

**Published:** 2025-11-19

**Authors:** Mark Grevsen Martinet, Bolaji John Samuel, Tinatini Tchatchiashvili, Daniel Weiss, Mathias W. Pletz, Oliwia Makarewicz

**Affiliations:** a Institute of Infectious Diseases and Infection Control, Jena University Hospital, Friedrich-Schiller University, Jena, Germany; b German Centre for Orthopaedics, Jena University Hospital, Friedrich-Schiller University, 07607, Eisenberg, Germany; c Leibniz Center for Photonics in Infection Research, Jena University Hospital, Friedrich-Schiller University, Jena, Germany

**Keywords:** Biofilms, Temperate bacteriophages, Pf1-like, Antibiotic resistance, Colistin, Tobramycin

## Abstract

Given the high resistance levels in Gram-negative bacteria, phage therapy is receiving increasing attention. In Germany, a clinical study is already underway evaluate a phage cocktail for treating *Pseudomonas aeruginosa* in cystic fibrosis (CF) patients. In this context, we investigated the prevalence of PF1-like prophages in *P. aeruginosa* isolates from a local CF cohort, their ability to undergo lytic conversion during biofilm formation, and the resulting impact on the resistance profile of the *P. aeruginosa* population. Consistent with other studies, prophage Pf4 was the most prevalent in this cohort and became during biofilm formation even in the absence of external triggers. This lytic conversion rapidly generated a subpopulation resistant to the virulent phages, potentially complicating phage therapy. However, this subpopulation also became more susceptible to most antibiotics commonly used in CF, suggesting a potential therapeutic opportunity. Interestingly, this bacterial subset lost its susceptibility to colistin, an important inhaled antibiotic in CF, which could increase the risk of treatment failure. These findings underscore both the challenges and potential strategies for improving treatment outcomes in CF patients.

## 1 Introduction

Cystic fibrosis (CF) is an autosomal recessive genetic disease caused by mutations in the cystic fibrosis transmembrane conductance regulator (CFTR) gene. CF affects millions of people worldwide and manifests primarily by the production of thick, sticky mucus in the airways, which promotes life-threatening respiratory complications and chronic infections. This CF mucus creates an ideal niche for pathogens to colonise the lungs and persist while evading immune defences [1] In particular, infections with *Pseudomonas aeruginosa*—a highly adaptable and virulent bacterium—are associated with markedly increased mortality in CF patients [2]. Therefore, a key focus of CF therapy is the prevention of PA infections [3]. When *P. aeruginosa* is detected, aggressive treatment is initiated using multiple antibiotics administered both orally and via inhalation, although success is not always achieved. A major factor contributing to the persistence and high resistance of *P*. *aeruginosa* is its ability to form biofilms, which shield. The embedded bacteria from antibiotics and the host immune response.

Additionally, the bacteria can transition into metabolically inactive forms, enabling them to persist for prolonged periods and cause recurrent infections [4,5]. According to the Cystic Fibrosis Foundation (https://www.cff.org), approximately 60 % of adults with CF suffer from infections or chronic colonisation of the lung by *P. aeruginosa*. This underscores the urgent need for alternative therapies that specifically target *P. aeruginosa* and its biofilms.

Bacteriophages (thereafter named phages) are viruses that specifically infect and kill bacterial cells. Virulent phages replicate immediately upon infecting bacteria leading to their lysis. These are considered potent therapeutics for difficult-to-treat, antibiotic-resistant bacterial infections. Phage therapy offers a precision medicine approach, in which tailored phage cocktails can be designed to target specific bacterial strains in individual CF patients [6]. In CF, phage therapy is already being explored in clinical trials. According to the ClinicalTrials.gov, five trials in CF-patients with phage-cocktails against *P. aeruginosa* are registered (as December 2023), with two phase 1/phase 2 trials being completed (NTC04596319 on AP-PA02, and NCT04684641 on YPT-01, both study results are still unpublished), a recruiting follow-up phase 2 trial on AP-PA02 (NTC05616221), another recruiting phase 1/phase 2 trial on WRAIR-PAM-CF1 (NCT05453578), and one active but currently non-recruiting trial on BX004-A (NTC05010577). These trials reflect the growing interest in phages as potent antimicrobials.

Temperate phages integrate into the bacterial genome as prophages, where they remain until reactivated. They are known to facilitate horizontal gene transfer [7] and are therefore less suitable as antimicrobial agents. Prophages are common in bacteria, particularly in *P. aeruginosa*. They can switch spontaneously or in response to various intracellular (e.g. DNA damage) or external (e.g., chemical stress) signals to the lytic cycle, producing and releasing new phage particles [8] This process is also referred to as lysogenic conversion. However, the interactions between therapeutically applied virulent phages and resident prophages remain poorly understood.

Prophages are increasingly recognised as key players in the complex interplay between host, pathogen and environment in CF [[9], [10], [11]]. Prophages can carry genes that confer various benefits to their bacterial host, including antibiotic resistance, virulence factors, and metabolic traits that enhance adaptation to environmental stress [12]. For instance, prophages may encode toxins and secretion systems that increase bacterium's pathogenicity, contributing to tissue damage and exacerbation of lung infections [13]. These genetic payloads can profoundly impact *P. aeruginosa's* capacity to colonise, persist, and resist immune clearance within the CF lung. Prophage induction can also promote biofilm formation, a critical factor in the chronicity of *P. aeruginosa* infections [14,15]. These interactions shape the clinical course of CF and present challenges for therapeutic management, particularly in the context of phage therapy. In this study, we analysed the prophage profiles of *P. aeruginosa* strains from a German CF cohort and investigated their ability to undergo lysogenic conversion and the resulting impact on antibiotic susceptibility and treatment with virulent phages.

## 2 Results and discussion

### 2.1 Antimicrobial resistance of the *P. aeruginosa* isolates

Demographic details of the CF cohort, including patient age, sex, and specimen distribution, are provided in the Supplementary Material (Fig. S2).

**Fig. 1:**
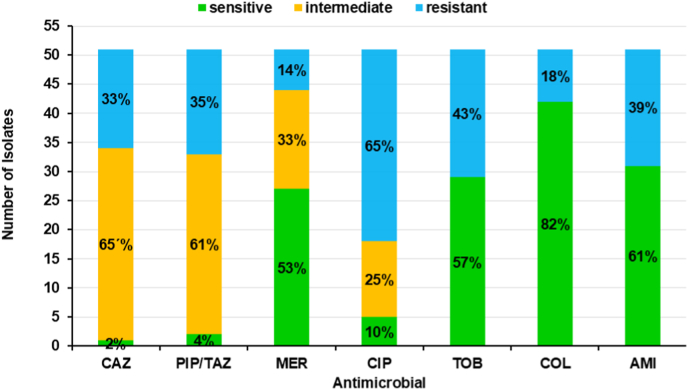
**Distribution of the antimicrobial susceptibility to antibiotics relevant in treatment of infections in CF- patients**. The values in the bars indicate the fraction of the resistant, susceptible and intermediate MICs. CAZ = ceftazidime, PIP/TAZ = piperacillin/tazobactam, MER = meropenem, CIP = ciprofloxacin, TOB = tobramycin, COL = colistin, AMI = amikacin.

Resistance profiles of the 51 *P. aeruginosa* isolates from the CF cohort were determined during routine diagnostics as minimal inhibitory concentration (MIC) values and interpreted according to EUCAST breakpoints as susceptible, intermediate, or resistant. Testing included the key anti-pseudomonal antibiotics ceftazidime (CAZ), piperacillin/tazobactam (PIP/TAZ), meropenem (MER), ciprofloxacin (CIP), tobramycin (TOB), colistin (COL), and amikacin (AMI) (Fig. 1).

Susceptibility rates were low for CAZ (2 %) and PIP/TAZ (4 %), with resistance rates of 33 % and 35 %, respectively; most remaining isolates showed intermediate susceptibility. For MER, 53 % of isolates were susceptible, 33 % intermediate, and 14 % resistant. CIP resistance was common (65 %), with only 10 % of isolates susceptible and 25 % intermediate. TOB susceptibility was 57 %, with 43 % resistant. The highest susceptibility was observed for COL (82 % susceptible, 18 % resistant). AMI showed 61 % susceptibility and 39 % resistance.

Overall, the data confirm the high prevalence of multidrug resistance in *P. aeruginosa* from CF patients, underscoring the therapeutic challenges in this population.

### 2.2 Distribution of the prophage Pf1-like specific genetic element

The Pf phages are the filamentous inoviruses that infect bacteria via type IV pili [14]. Various Pf1-like genetic elements can be found in *P. aeruginosa*. In a previuos PCR-screening study from 2015, 60 % of 241 analysed *P. aeruginosa* strains carried at least one genetic element of the Pf-1-like family, with Pf4 being most prevalent [16].

**Fig. 2:**
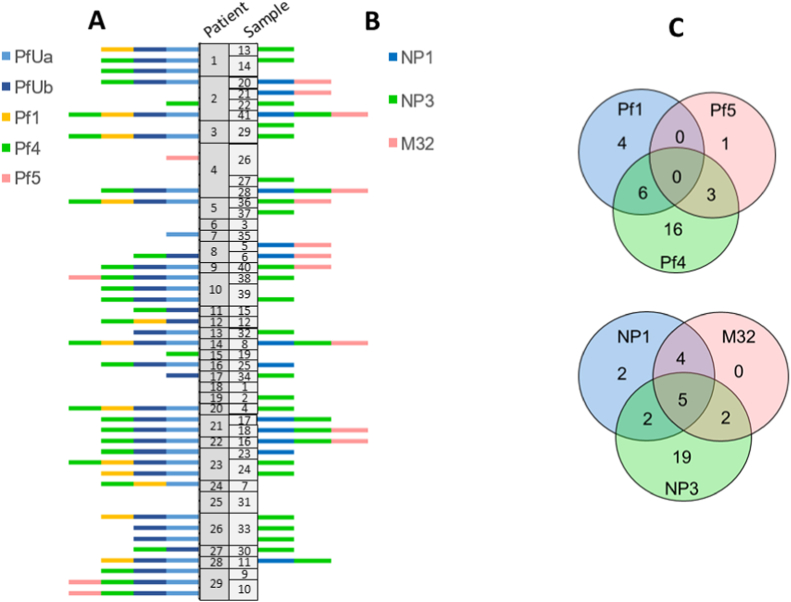
**Distribution of prophage-related genetic elements and susceptibility to virulent phages in the CF *P. aeruginosa* isolates**. (A) Presence of prophage amplicons detected using universal primers (PfUa and PfUb) or specific primers for Pf1, Pf4, and Pf5. (B) Susceptibility profiles to virulent phages NP1, NP3, and M32. The Y-axis indicates randomly assigned patient's ID and samples IDs, while the X-axis indicates the presence of the prophage amplicon (A) or susceptibility to virulent phage (B), as shown in the legend (colour coded). (C) Venn diagrams illustrating the overlap the distributions of the prophage elements and susceptibility to virulent phages among the CF isolates.

Using the previously described universal primers PfUa and PfUb [16], we screened the CF isolates for the presence of Pf1-like prophage signatures by PCR (Fig. 2 A). PfUa and PfUb amplicons were detected in 59 % and 65 % of the isolates, respectively, with 55 % positive for both PCR products. These frequencies were higher than those reported by Mooji et al. and Knezevic et al., where PfUa amplicons were detected in 44 % and 41 %, respectively, and PfUb in 52 % of strains from various sources, including environmental isolates [16,17].

We also used primers targeting specific genetic elements of Pf1-prophage subgroups Pf1, Pf4 and Pf5 [16,17]. Pf4-specific amplicons were the most common, found 55 % of isolates, followed by Pf1 in 24 %. Compared with Knezevic et al. [16] the prevalence of Pf1 was only slightly higher in our CF cohort (24 % vs. 17.8 %) and simultaneous presence of Pf4 with the general PfU and Pf1 amplicons did not significantly. However, the frequency of Pf4-specific amplicons in our cohort was more than twice as high, which explains the higher prevalence of PfU amplicons overall and suggests a possible enrichment of specific *P. aeruginosa* subtypes in CF patients. Pf1 is the only known Pf1-like prophage capable of persisting extrachromosomally [18], whereas Pf4 has been linked to increased virulence of *P. aeruginosa* in a mouse model [19].

In agreement with Knezevic et al. [16], Pf5-specific amplicons were rare, detected in only four isolates (7.8 % vs 6.6 %), and no isolate carried signatures of all three prophages (Fig. 2C). Only 7.8 % of isolates (n = 4) were positive solely for the universal primers, while 29 % (n = 15) were negative for both PfUa and PfUb amplicons. Furthermore, in 24 % (n = 12) none of the specific prophage fragments (Pf1, Pf4, Pf5) amplified (compared to 39.8 % in Knezevic et al.). PfUa and PfUb signatures correlated well with each other but showed only weak positive correlation with the specific Pf1 and Pf4 amplicons, and almost no correlation to Pf5 (Pearsons's rank (r_p_) = 0.71, Supplementary Material Fig. S3). This suggests that the universal primes might not fully capture the diversity of Pf1-like prophages in the CF cohort. The absence of PfU amplicons could also indicate the presence of Pf7 phage clade, as previously proposed [16] or simply result from genetic alterations in the CF-isolates.

A recent study in Standfort and Danish cohorts found that the isolates from older CF patients were more likely to be Pf-positive [20]. In our cohort, however, we observed only a weak positive trend between the presence of Pf1 amplicons and patient age.

### 2.3 Susceptibility to the virulent phages NP1, NP3 and M32

The CF-isolates were tested for their susceptibility to the three virulent phages NP1, NP3, and M32, which exhibit broad host ranges against *P. aeruginosa* [21,22] and are therefore considered promising candidates for phage therapy in CF patients. We assessed each isolate's response to these phages (Fig. 2 B) and classified the outcomes as susceptible, reduced susceptibility, or resistant.

Was observed in 25 % of isolates for NP1, 55 % for NP3, and 22 % for M32, while resistance was detected in 67 %, 35 %, and 73 % of isolates, respectively. The remaining isolates showed reduced susceptibility, evident on agar plates as isolated colony growth with the lysis zones. Since reduced susceptibility may led to treatment failure in phage therapy, we proposed that this phenotype should be interpreted as resistance for diagnostic purposes.

Susceptibility to all three virulent phages was observed in 33 % (n = 17) of the CF isolates, whereas 10 % displayed reduced susceptibility or resistance to all three. Susceptibility to NP3 alone was found in 37 % of isolates, and to NP1 alone in 3.9 %, while no isolate was susceptible solely to M32 (Fig. 2C). These results indicate a generally high level of resistance among CF *P. aeruginosa* strains to these virulent phages, which could lead to treatment failure in phage therapy applications.

Despite belonging to different phage classes, resistance to NP1 and M32 correlated positively (Supplementary Material Fig. S3), suggesting that they utilise the same bacterial receptor for infection. It might be interesting to analyse if these phages also compete when used together in a cocktail resulting in antagonism and reduced effectivity [23].

### 2.4 Correlation between prophages, and susceptibilities to phages and antimicrobials

No clear correlations were observed between resistance to the virulent phages and the presence of the prophages, although some trends could be noted. However, interpretation of these findings is challenging, as comparable analyses are scare in other descriptive studies.

A negative correlation trend (r_P_) was observed between Pf1 and NP3 (Supplementary Material Fig. S3), while the presence of Pf5 elements showed low but positive r_P_ values. Given the low prevalence of Pf1 and Pf5 in our cohort, analysis of a larger number of isolates would be valuable to determine whether Pf1 presence could serve as an indicator of increased susceptibility, and Pf5 as a potential marker of reduced susceptibility, to specific virulent phages used therapeutically.

Additionally, weak negative trends were observed between the presence of Pf1 signatures and resistance to MER, CIP, and COL. Resistance to NP1 and M32 showed negative correlation trends with all three β-lactam antibiotics tested (CAZ, PIP/TAZ, and MER).

### 2.5 Clonality of the isolates

Although we did not sequence the isolates, our findings align with the well-documented variability of *P. aeruginosa* populations in the CF lung [24]. Notably, isolates from the same patient—whether collected at different time points or from the same specimen—can differ greatly in their properties. Even when the prophage pattern was similar, particularly among isolates from a single specimen, their susceptibility to virulent phages and their resistance profiles often differed. This variability highlights the complexity of managing CF lung infections patients, as the differences in phage susceptibility and antibiotic resistance can substantially influence efficacy and clinical outcomes.

### 2.6 Prophage induction in *P. aeruginosa* biofilms

Bacterial biofilms in chronic infections are subject to nutrient limitations and hypoxia, which contribute to their antibiotic tolerance and persistence. Studies have shown that oxygen gradients within biofilms can reach extremely low levels, creating hypoxic conditions [25]. Hypoxia, nutrient limitations, and host immune responses can all stimulate prophage induction, leading to bursts of phage production and bacterial cell lysis [26]. Therefore, we studied the Pf4 phage reactivation during biofilms formation and maturation in the laboratory strain PAO1 and the three clinical isolates (CF-PA-6, CF-PA75 and CF-PA83). CF-PA 75 and CF-PA 83 were selected because they both carried Pf4 signatures and were susceptible to all three exogenous phages. The clinical isolate CF-PA83 was also positive for Pf1, while PAO1 and CF-PA75 were both Pf1-negative in the PCR. Isolate CF-PA6, which lacked Pf1-like prophage signatures, served as a control.

Presence of phages with lytic activity in biofilm supernatants was assessed daily by the spot tests against the corresponding host strain (self-infectivity) and the presence of reactivated phages was confirmed by PCR for the supernatants after 5 days of growth (Supplementary Material, Fig. S4). Pf1 was not detected in the supernatants of any of the strains, whereas Pf4 was detected in all supernatants except that of CF-PA6. Pf5 was additionally identified in the supernatant of CF-PA83.

**Fig. 3:**
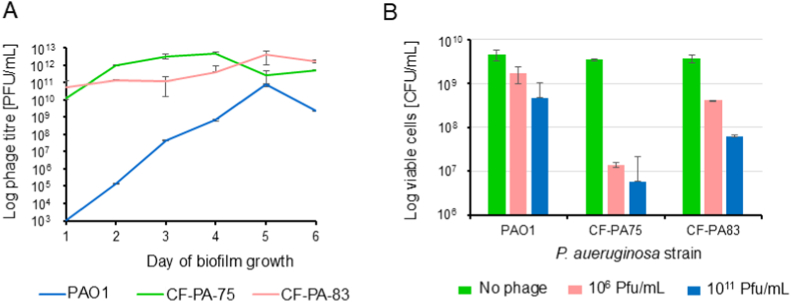
**Pf4 induction and infectivity in biofilms of selected clinical isolates and PAO1. (**A) Lytic activity of the supernatant across several days of biofilm growth. The phage titer (PFU/mL) was assessed in PAO1 for all supernatants (see legend). (B) Self-infectivity of the induced temperate phages on 24 h old biofilms of PAO1, CF-PA75 and CF-PA83 determined as viable bacteria (CFU/mL) after 16–18 h of phage treatment. Two different phage concentrations (10^6^ and 10^11^ PFU/mL) were tested.

Phage titres in biofilm supernatants were quantified over time during biofilm maturation in PAO1 (Fig. 3A). In the laboratory strain PAO1, Pf4 titres increased steadily, peaking after five days before slightly declining on day six. In contrast, supernatants from clinical isolates showed high phage titres after just one day of incubation, which also increased by two orders of magnitudes over time. The higher initial titres in clinical isolates compared to PAO1 may reflect their generally greater cross-infectivity, whereas PAO1 showed a more gradual, time-dependent increase in self-infectivity. This phenomenon was not investigated in detail here. The strong cross-activity of clinical isolate supernatants against PAO1, evident after only one day of biofilm growth, indicates that prophages in CF isolates can be readily reactivated and released. Given that Pf4 has been associated with increased *P. aeruginosa* virulence [21], such lysogenic conversion events could potentially exacerbate lung disease in CF patients.

We further tested the self-infectivity in 24-h-old biofilms by each strain overnight (16–18 h) with two concentrations the phage lysates (10^6^ and 10^11^ PFU/mL) obtained from the PEG enrichment. After treatment, biofilms were washed, resuspended in fresh LB broth, and plated on LB agar to determine viable bacterial counts as CFU/mL (Fig. 3B). Phage lysates reduced the bacteria number in a concentration-dependent, though not strictly linear, manner. In PAO1 biofilms, Pf4 showed lower virulence, with 10^11^ PFU/mL achieving only a one-log reduction. In CF-PA75, Pf4 exhibited the highest activity, producing a two-log reduction at 10^6^ PFU/mL, with an additional one-log decrease at 10^11^ PFU/mL. In CF-PA83, bacterial counts decreased stepwise by one log at each concentration. As CF-PA83 harbours both Pf4 and Pf5, the observed effect cannot be attributed exclusively to Pf4; reduced efficacy may result from competitive interactions between the two phages, as both target the type IV pilus.

Prophage carriage typically confers immunity to infection by related phages [26]. Therefore, the observed self-infection indicates that Pf4 (and possibly Pf5) can undergo superinfection. The nonlinear relationship between PFU/mL and CFU/mL may reflect an interplay between superinfectivity of reactivated temperate phages and the development of phage resistance mechanisms within biofilms. Superinfection suggests that Pf4 and Pf5 may acquire genetic alterations during reactivation, enabling them to bypass host immunity. Indeed, superinfective induced prophages have been reported to display an increased mutation frequency, which can drive an irreversible switch from a non-lytic phenotype—such as the filamentous Pf4 phage—to a lytic phenotype [24].

It should be noted that even when the phage concentration used for reinfection was nearly two logs higher than the initial bacterial count, complete elimination of the biofilm bacteria was not achieved. Approximately 10 % of the PAO1 population, 1 % of CF-PA83, and 0.1 % of CF-PA75 remained unaffected by the reactivated phage, indicating that these subpopulations could persist over time. Whether this persistence is due to newly acquired bacterial resistance mechanisms or to the integrated prophage restoring immunity to reinfection remains unclear. These questions will be addressed in future studies.

We investigated the potential transmission of the Pf4 prophage from CF-PA73 to CF-PA6 by co-culturing the strains in a mixed biofilm for six days. However, no Pf4-specific amplicons were detected in CF-PA6 after co-cultivation, indicating that transmission did not occur under the tested conditions. This suggests that CF-PA6 may be either resistant or inherently unsusceptible to infection by Pf4-like phages.

### 2.7 Resistance to virulent phages after biofilm formation

Superinfection can drive the bacterial evolution and increase genetic diversity, potentially leading to resistance aganst other phages [27]. Therefore, we further investigated whether reactivation of Pf4 (*Inovirdae*) influences bacterial susceptibility to the unrelated virulent phages NP1 (*Siphoviridae*), NP3 (*Myoviridae*) and M32 (*Podoviridae*).

**Fig. 4:**
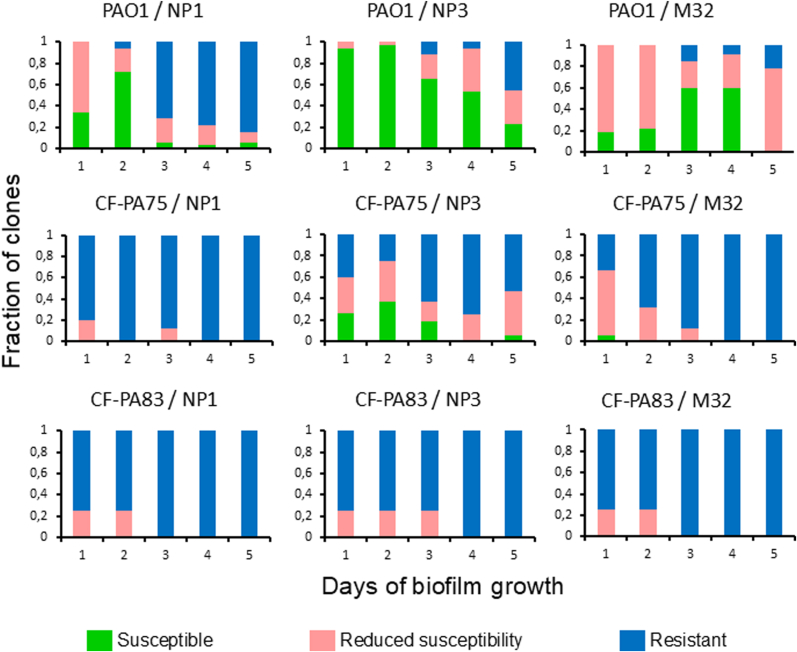
**Development of resistance to virulent phages in selected clinical isolates and PAO1.** Resistance profiles to virulent phages NP1, NP3 and M32 were assessed for biofilm-embedded clones (daily n = 32 colonies randomly selected) in time-dependent manner.

Biofilms grown from 1 to 5 days were resuspended in fresh LB broth after removal of the supernatants. After serial dilution, the bacteria were plated on agar, and randomly selected colonies (in total 32) were tested in a cross-streak agar assay with the virulent phages NP1, NP3 and M32 to assess the proportion of susceptible and resistant clones. Clones showing reduced sensitivity, evidenced by individual colonies growing beyond the phage inoculation line, were also recorded (Fig. 4).

All bacterial strains (PA01, CF-PA75 and CF-PA83) were susceptible to the three virulent exogenous phages prior biofilm formation. After just one day of biofilm formation, a substantial fraction of PAO1 clones already showed reduced susceptibility to NP1 and M32, while the clinical isolates displayed high proportions of resistant clones to all three phages. Overall, PAO1 biofilms retained the highest levels of susceptibility, particularly to NP3. N CF-PA75, NP3-susceptible fractions persisted even in clones from 5-day-old biofilms. In all strains, resistance fractions increased over time and against all phages, but this shift occurred notably faster in the clinical isolates. By day three or four, most clones were resistant to the virulent phages, especially NP1 and M32.

### 2.8 Changes in antibiotic resistance profiles after prophage induction

**Fig. 5:**
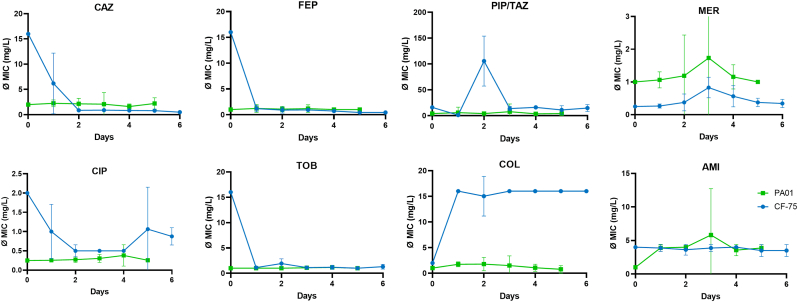
The effect of lysogenic conversion of the temperate bacteriophages on the susceptibility towards antibiotics. The y-axes represent the corresponding antimicrobial's minimal inhibitory concentration (MIC) (as indicated above each graph). X-axes indicate the growth of the biofilm. The MICs were assessed for 32 clones for every day of biofilm growth and shown as mean and standard deviation. CAZ, ceftazidime; FEP, cefepime; PIP/TAZ, piperacillin/tazobactam; MER, meropenem; CIP, ciprofloxacin; TOB, tobramycin, COL, colistin; AMI, amikacin.

Changes in antibiotic susceptibility during biofilm growth over 5 days were monitored daily. Each day, 32 colonies grown from the resuspended biofilms of PAO1 and CF-PA75 were randomly subjected to antimicrobial susceptibility testing (AST). The antibiotics used in this analysis included cephalosporins (ceftazidime (CAZ), cefepime (FEP)), meropenem (MER, a carbapenem), broad-spectrum piperacillin (PIP) combined with a β-lactamase inhibitor tazobactam (TAZ), ciprofloxacin (CIP, a fluoroquinolone), two aminoglycosides - tobramycin (TOB) and amikacin (AMI), and colistin (COL, a polymyxin) (Fig. 5).

The original bacterial strain, PAO1 (time point 0), was susceptible to all antibiotics tested. Clones obtained from biofilms showed increased MICs for β-lactams and CIP in selected colonies, most notably at day 3 of biofilm growth. This point coincided with the first detectable phage transmissions in the previously prophage-free recipient in mixed biofilms. We therefore hypothesis that the observed MIC increases in PAO1 may be linked to prophage induction and superinfection. However, all MIC changes remained below resistance breakpoints.

In contrast, the clinical isolate CF-PA75 exhibited MICs above resistance breakpoints for several antibiotics already at baseline, including 128 mg/L for PIP/TAZ, 16 mg/L each for CAZ, ATC and FEP, and 2 mg/L for CIP During biofilm maturation, MICs for carbapenems increased slightly, with imipenem (IMI) occasionally exceeding the clinical breakpoint (4 mg/L) in selected colonies Interestingly, MIC values for cephalosporins, CIP, and aminoglycosides (except AMI, which remained low throughout) declined over time, and most colonies became susceptible during the last two days of biofilm growth. The most pronounced effect was seen for COL, where MICs increased sharply after only one day, reaching 16 mg/L in most colonies regardless of biofilm age.

We hypothesise that these changes in antibiotic susceptibility are linked to mechanisms of phage resistance, as resistance to virulent phages in the CF-PA75 biofilm population emerged after the first day of biofilm formation. In our recent study, in which biofilms of various laboratory and clinical *P. aeruginosa* strains were sequentially exposed to NP3, NP1, or M32 phages, we identified mutations in structural genes of the type IV pilus machinery that were frequently associated with phage resistance [28]. While type IV pili are primarily involved in adherence, motility, and uptake of free DNA, it remains unclear how such mutations might influence susceptibility to different antibiotic classes. Another possibility is alterations in lipopolysaccharide (LPS) structure. In the same study, some phage-resistant clones carried mutations in LPS synthesis genes, which could explain the elevated COL MICs, as COL targets bacterial membranes through LPS interactions [29]. Such changes could reduce COL's ability to destabilise and permeabilise the bacterial membrane. However, the mechanisms by which these alterations might also impact the efficacy of β-lactams, CIP, and aminoglycosides remain unclear.

### 2.9 Statistical analysis of MIC changes

Statistical analysis of MIC changes during biofilm growth revealed pronounced strain-specific and antibiotic-class-dependent patterns (Supplementary Material Table S3). In the patient isolate CF-75, significant temporal differences (*p* < 0.05) were detected for nearly all antibiotic classes except for the carbapenems MER and IMI, which remained stable throughout biofilm maturation. β-Lactams, including PIP/TAZ, CAZ, FEP, and ATC, showed strong and consistent changes, with significant increases already apparent on day 1 and persisting thereafter. Similar dynamics were observed for the aminoglycosides AMI and TOB and for the polymyxin COL, all exhibiting highly significant time-dependent MIC shifts (*p* < 0.001). In contrast, the laboratory strain PAO1 showed a much less responsive profile, with AMI remaining the only antibiotic demonstrating consistent differences over time.

The divergence between PAO1 and CF-75 likely reflects fundamental differences in genetic flexibility and adaptive potential. CF-75, a clinical isolate chronically exposed to the fluctuating and selective conditions of the cystic fibrosis lung, appears to rapidly adjust its antimicrobial susceptibility during biofilm growth, consistent with prophage-mediated and regulatory plasticity. By contrast, the long-domesticated PAO1 strain, adapted to stable laboratory conditions, exhibited limited responsiveness. The absence of statistical significance in many PAO1 comparisons mainly results from the high variation among individual colonies (large standard deviations), reflecting heterogeneous clonal behaviour rather than a lack of biological change. These observations underscore that biofilm-associated adaptation and prophage activation exert a far greater phenotypic impact in genetically flexible clinical isolates than in stable laboratory lineages.

### 2.10 Conclusions

The Pf1-like prophage family (*Inoviridae*) is a well-known and highly prevalent family in *P. aeruginosa* strains. Our investigation into the presence and dynamics of PF1-like prophages in *P. aeruginosa* isolates from CF patients has uncovered significant challenges and promising therapeutic opportunities. The predominance of the temperate phage Pf4 and its ability to undergo lysogenic conversion during biofilm formation play a crucial role in shaping the population dynamics within the CF lung. The superinfective virions can potentially transmit to other prophage-free isolates, complicating the population structure. This could not be proved in the present studies, but it cannot be excluded.

One critical finding is the reduced susceptibility of a sub-population to virulent phages following prophage reactivation, which may pose a challenge to the efficacy of phage therapy. However, concurrently restoring susceptibility to most CF-relevant antibiotics in this sub-population offers a novel therapeutic avenue. Targeted prophage reactivation could sensitise multidrug-resistant *P. aeruginosa*, potentially enhancing or restoring the effectiveness of conventional antibiotics.

An important consideration is the observed loss of colistin susceptibility in the sub-population post-reactivation. This could lead to treatment failure when colistin is used as an inhaled antipseudomonal antibiotic, necessitating careful management and alternative therapeutic strategies.

Overall, our findings provide valuable insights into the complex interactions between prophages and virulent phages and antibiotic susceptibility in *P. aeruginosa*, presenting both hurdles and opportunities for improving the treatment outcomes of CF patients. Hypoxia in CF lung biofilms may influence prophage induction, and experiments under more physiologically relevant conditions could help clarify prophage–host dynamics in this environment. Further research is warranted to explore the mechanisms underlying these interactions and to develop targeted strategies for optimising CF therapy.

## 3 Materials and methods

### 3.1 Bacterial strains

This study examined 51 *P. aeruginosa* isolates from CF patients retrospectively (see also Supplementary Material Table S1). These originated from sputum, nasal lavage, and throat swap of CF patients collected at the Cystic Fibrosis Center for Children and Adults (Jena University Hospital, Germany) during the regular check-ups or acute symptoms of the patients. The *P. aeruginosa* strains were routinely isolated and confirmed by matrix-assisted laser desorption ionisation time-of-flight (MALDI-TOF) (Brucker Daltonics, Billerica, US) and the resistograms were performed using VITEK®2 (bioMerieux, Marcy l' Etoile, France) at the Institute of Medical Microbiology (Jena University Hospital, Germany). Where MIC values were missing, they were determined using the microdilution method as recommended by EUCAST. MIC values falling between the EUCAST-defined clinical breakpoints for susceptibility and resistance were interpreted as intermediate in this study. The retrospective use of the isolates and selected pseudonymized data of the patients, was approved by the ethics committee of the Jena University Hospital (Germany) under the registration number 2023-3199-Material.

Laboratory *P. aeruginosa* strain PAO1 was used as a control in several experiments, and to proliferate virulent phages and titrate the temperate and virulent phages.

### 3.2 Phages

Three different virulent exogenous phages were used: NP1 phage (family *Siphoviridae*, *Nipunavirus*, GenBank: KX129925.1) with lytic activity against PA14 but not against PAO1 as well as NP3 phage (family *Myoviridae, Pbunavirus*, GenBank: KU198331.1), showing a broad range lytic activity against different *P. aeruginosa* strains were initially isolated by Chaudhry et al., from a local sewage treatment plant [21]; M32 phage (family *Autographiviridae,* Phikmvvirus,*G*enBank: KX711710.1) with lytic activity against PAO1 but not PA14, was isolated by Karumidze et al. from a Georgian sewage water [22].

### 3.3 Culture conditions of bacteria

The *P. aeruginosa* strains were cultured on Columbia blood agar or cetrimide agar (all bioMerieux). Single colonies were cultured overnight (approximately 16 h) in a 4 mL Luria-Bertani (LB) broth (Carl Roth GmbH, Karlsruhe, Germany) at 37 °C with agitation of 160 rounds per minute (rpm) on an orbital shaker GFL 3032 (GFL Gesellschaft für Labortechnik GmbH, Burgwedel, Germany). These overnight cultures were used to prepare the experiments and cryo-stocks with 10 % glycerol (V/V), which were stored at −80 °C.

To grow biofilm, the overnight cultures were adjusted to an optical density (OD_600_) of 0.08 and grown in 96-well microtiter plates (Sarstedt AG &Co.KG, Nürnbrecht, Germany) in LB broth for several days (see specific methods below) under static conditions at 37 °C.

To determine viable bacteria in the biofilms, the biofilms were washed twice carefully with 0.9 % NaCl and the bacteria were resolved in fresh LB broth by scraping the biofilms from the flat-bottom 96-well titer plates. The bacterial suspension was serially diluted, and 100 μl of selected dilutions were spread on LB agar, and grown overnight at 37 °C.

### 3.4 Phage proliferation and titration

Phage proliferation was performed using PA14 as the host for NP1 and NP3, and PAO1 as the host for M32. The bacteria were grown in LB broth until log-phase (OD_600_ 0.5–0.8), adjusted to OD_600_ of 0.1 and 0.3 mL, mixed with 0.1 mL of phage lysate suspension, and incubated for 30 min at 37 °C. Subsequently, 4 mL of LB top agar (0.5 %) was added, and the phage-bacteria was overlayed on the LB-agar plate (1.2 %) and incubated overnight at 37 °C. To recover the phages, 5 mL of LB broth was poured over a plate with confluent but separate plaques and incubated for 2 h with shaking at 80 rpm. The medium was transferred to 1.5 ml tubes and centrifuged. The supernatants were combined and filtered through a 0.2 μm syringe filter (Merck KGaA, Darmstadt, Germany) to obtain crude phage lysate and stored at 4 °C. The same procedure was applied to determine the phage titre, but the phage lysates were diluted logarithmically before mixing with bacteria, and the plaques were counted after incubation.

### 3.5 Isolation of induced prophage in biofilms

Strain PAO1 and clinical isolates CF-AP6, CF-PA75 and CF-PA83 were grown in LB broth without shaking for up to 4 days to form biofilms and supernatants were collected on the last day. The supernatants were centrifuged at 4000 g for 30 min at 4 °C and filtered with a 0.45 μm syringe filter (Merck Milipore, Burlington, USA) and treated by DNase I (1.25 U) (ThermoFischer Scientific) for 1 h at 37 °C to remove free DNA. Subsequently, the DNase was inactivated 75 °C for 10 min.

### 3.6 Phage spot test and cross-streak agar assay

This test was used to qualitatively determine lytic activity in the supernatants of the induced prophages on its host strain (self-infectivity) or another bacterial strain (cross-infectivity). A fresh bacterial culture was adjusted to an OD_600_ of 0.1, and 0.3 mL of the bacterial suspension was mixed with 5 mL of top agar (0.5 % agar-gar in LB) and poured onto an LB plate. After solidification, 10 μL of the lysate was spotted (two spots each) onto the plate and incubated overnight. The presence of clear plaques/halos indicated the susceptibility, while single colonies within the halo indicated a resistant/tolerant subpopulation.

### 3.7 Transmission of Pf4 in mixed biofilms

Overnight cultures of CF-PA75 and CF-PA6 were adjusted to OD_600_ of 0.08, mixed to equal volumes in 96-well microtiter plates (Sarstedt AG &Co.KG), and grown under static conditions at 37 °C up to 5 days to form a mixed biofilm. The viable bacteria were assessed daily as described previously by selection for CF-PA75 and for CF-PA6 on LB agar supplemented with meropenem (8 g/L to select) or tobramycin (6 mg/L to select), respectively. Five randomly selected colonies were taken from each strain and time point, resolved in 100 μl sterile water and boiled for 10 min. After centrifugation for 10 min at 10,000 g and room temperature (RT), the presence of the Pf4 phage was assessed in the supernatants by specific PCR.

### 3.8 Amplification of Pf1-like prophage elements

To determine prophage-related genetic elements, previously described primers (each 200 nM) listed in Supplementary Material Table S2 (18,19), 100 ng of the genomic bacterial DNA and the Dream Taq DNA polymerase kit (ThermoFischer Scientific) were mixed according to the manufacturer's recommendation in 20 μl volume. The PCR was performed in the Veriti 96 well thermo-cycler (Applied Biosystems, Waltham, USA) under the following conditions: an initial denaturation steps of 95 °C for 2 min, followed by 40 cycles of denaturation (95 °C, 3 s), annealing (62 °C, 2 s) extension (72 °C, 1 min). DNA Gel Loading Dye ( × 6) was used to prepare the samples, and O'GeneRuler DNA ladder was used as a marker (both ThermoFischer Scientific). The amplicons were separated by electrophoresis on 2 % agarose gels supplemented with ethidium bromide (0.2 μg/mL) at 100 V and documented using G-Box F3 (Syngene, Cambridge, UK).

### 3.9 Antibiotic resistance assay via VITEK 2

We investigated the resistance pattern of randomly selected clones (each strain and treatment N = 32) isolated from biofilm after prophage induction for their susceptibility against several antibiotics. Individual wells were streaked out on an LB agar plate and incubated overnight. The selected colonies were resuspended each in 0.45 % NaCl and adjusted to an OD600 0.50–0.63. The antimicrobial susceptibility profiles were assessed by VITEK®-2 system using the cartridges AST-N389 and AST-N232 for gram-negative organisms (all bioMérieux, Marcy-l’Étoile, France) according to the manufacturer's instructions.

### 3.10 Software and statistical analysis

Microsoft Excel (Microsoft Corporation, Redmond, USA) and GraphPad Prism 9 (GraphPad Software Inc., San Diego, USA) were used for calculations, analyses and visualization of experimental data. Statistical analysis of the MICs was performed using two-way ANOVA followed by Tukey's multiple-comparison test. The analysis was conducted separately for each strain and antibiotic to assess temporal differences during biofilm growth. Significant pairwise differences are summarized in Supplementary Material Table S3.

Pearson correlation coefficients (*rP*) were calculated in Excel. As Excel's Pearson function does not provide p-values, correlations were used in an exploratory manner to indicate potential relationships rather than to perform formal hypothesis testing.

## CRediT authorship contribution statement

**Mark Grevsen Martinet:** Writing – review & editing, Writing – original draft, Methodology, Formal analysis, Conceptualization. **Bolaji John Samuel:** Investigation. **Tinatini Tchatchiashvili:** Methodology, Investigation, Formal analysis. **Daniel Weiss:** Methodology. **Mathias W. Pletz:** Funding acquisition. **Oliwia Makarewicz:** Writing – review & editing, Supervision, Formal analysis, Conceptualization.

## Funding

This project has received funding from the European Union's Horizon 2020 research and innovation program under the Marie Skłodowska-Curie grant agreement No 861323, and from the Federal Ministry of Research, Technology and Space (BMFTR, Germany) grand number 13N15720.

## Declaration of competing interest

The authors declare the following financial interests/personal relationships which may be considered as potential competing interests: Mark Grevsen Martinet reports financial support was provided by European Commission Marie Sklodowska-Curie Actions. Mathias W. Pletz reports financial support was provided by Federal Ministry of Research, Technology and Space. If there are other authors, they declare that they have no known competing financial interests or personal relationships that could have appeared to influence the work reported in this paper.

## Data Availability

Data will be made available on request.

## Appendix A Supplementary data

Mutimedia component 1 Table S1 Patient cohort used in this study, Table S2 Prophage primers used in this study, Table S3 Significant pairwise differences (p-values for origin vs. day *n*) in minimal inhibitory concentrations (MICs) during biofilm growth. Fig. S1 Exemplary test of cross-streak agar assa., Fig. S2 Range of the patients' age included in this study. Figure S3 Figure S3 Pearson correlation matrix of the relevant isolates' properties. Fig. S4 Phage detection in the supernatants of biofilms of selected strains. **Additional results** on **Demographics of the CF cohort** and **Resistances of the *P. aeruginosa* strains**. Mutimedia component 1
