# Supplementary material for: Temperate bacteriophage induced in Pseudomonas aeruginosa biofilms can modulate bacteriophage and antibiotic resistance
Source: Biofilm. 2025 Nov 19;10:100333. doi: 10.1016/j.bioflm.2025.100333 (PMC12702240; doi:10.1016/j.bioflm.2025.100333)
Supplement: Mutimedia component 1 — Table S1 Patient cohort used in this study, Table S2 Prophage primers used in this study, Table S3 Significant pairwise differences (p-values for origin vs. day n) in minimal inhibitory concentrations (MICs) during biofilm growth. Fig. S1 Exemplary test of cross-streak agar assa., Fig. S2 Range of the patients' age included in this study. Figure S3 Figure S3 Pearson correlation matrix of the relevant isolates' properties. Fig. S4 Phage detection in the supernatants of biofilms of selected strains. Additional results on Demographics of the CF cohort and Resistances of the P. aeruginosa strains. [file mmc1.pdf]

Supplementary Material: Additional Details for Materials, Methods and Results

Temperate bacteriophages induced in *Pseudomonas aeruginosa* biofilms can modulate bacteriophage and antibiotic resistance

Authors: Mark Grevsen Martinet[1], Bolaji John Samuel[1,4], Daniel Weiss[1], Tinatini Tchatchiashvili[1], Mathias W. Pletz[1,5]; and Oliwia Makarewicz[1,5*]

[1] Institute of Infectious Diseases and Infection Control, Jena University Hospital/ Friedrich-Schiller University, Jena, Germany

[2] German Cancer Consortium (DKTK), partner site Frankfurt, a partnership between DKFZ and University Hospital Frankfurt, Frankfurt am Main, Germany.

[3] Goethe University Frankfurt, University Hospital Frankfurt, Department II of Internal Medicine, Infectious Diseases, Frankfurt am Main, Germany

[4] Department of Orthopedics, Jena University Hospital, Eisenberg, Germany.

[5] Leibniz Center for Photonics in Infection Research, Jena University Hospital/ Friedrich-Schiller University, Jena, Germany

Table S1 Patient cohort used in this study

ID Patient	Age	Sex	ID Specimen	Material	Study number	Mucoidity of PA	Other pathogens
1	37	Male	13	Throat swap	MCFJ101		/
1	37	Male	14	Throat swap	MCFJ3		/
1	37	Male	14	Throat swap	MCFJ2		/
2	36	Male	20	Nasal lavage	MCFJ4	Mucoid	/
2	48	Male	21	Sputum	MCFJ5	Mucoid	SA
2	24	Male	22	Sputum	MCFJ36	Mucoid	/
2	42	Male	41	Nasal lavage	MCFJ83	Mucoid	SA
3	20	Male	29	Throat swap	MCFJ6		/
3	45	Female	29	Throat swap	MCFJ7		/
4	39	Female	26	Sputum	MCFJ10		SA
4	39	Female	26	Sputum	MCFJ8		SA
4	39	Female	26	Sputum	MCFJ9	Mucoid	SA
4	39	Female	27	Sputum	MCFJ11		NF
4	39	Female	28	Sputum	MCFJ20		NF
5	18	Male	36	Sputum	MCFJ84	Mucoid	NF
5	42	Male	37	Sputum	MCFJ12	Mucoid	SA
6	7	Female	3	Throat swap	MCFJ13		/
7	17	Female	35	Throat swap	MCFJ15		SA
8	36	Male	5	Nasal lavage	MCFJ31	Mucoid	SA
8	25	Male	6	Sputum	MCFJ30	Mucoid	/
9	8	Female	40	Throat swap	MCFJ24		SA
10	20	Female	38	Sputum	MCFJ25	Mucoid	SA
10	36	Female	39	Sputum	MCFJ58	Mucoid	SA
10	20	Female	39	Sputum	MCFJ66	Mucoid	SA
11	37	Female	15	Nasal lavage	MCFJ28	Mucoid	/

12	36	Female	12	Throat swap	MCFJ38		SA
13	12	Female	32	Throat swap	MCFJ37		SA
14	36	Male	8	Sputum	MCFJ82	Mucoid	NF
15	20	Male	19	Sputum	MCFJ51		/
16	13	Female	25	Throat swap	MCFJ100		/
17	36	Female	34	Sputum	MCFJ68	Mucoid	SA
18	31	Male	1	Sputum	MCFJ67	Mucoid	/
19	33	Male	2	Throat swap	MCFJ71	Mucoid	NF
20	25	Male	4	Sputum	MCFJ72	Mucoid	NF
21	25	Male	17	Throat swap	MCFJ94		/
21	35	Male	18	Nasal lavage	MCFJ73	Mucoid	/
22	39	Female	16	Throat swap	MCFJ75	Mucoid	EC
23	25	Female	23	Sputum	MCFJ93		AF
23	57	Female	24	Sputum	MCFJ80		/
23	35	Female	24	Sputum	MCFJ81	Mucoid	/
24	51	Male	7	Nasal lavage	MCFJ85		NF
25	38	Male	31	Sputum	MCFJ87	Mucoid	NF
25	51	Male	31	Sputum	MCFJ86		NF
26	38	Male	33	Throat swap	MCFJ88	Mucoid	CA
26	39	Male	33	Throat swap	MCFJ89		CA
26	42	Male	33	Throat swap	MCFJ90		CA
27	15	Male	30	Sputum	MCFJ91		NF
28	57	Male	11	Throat swap	MCFJ92		NF
29	44	Female	9	Nasal lavage	MCFJ99		/
29	44	Female	10	Sputum	MCFJ96	Mucoid	/
29	44	Female	10	Sputum	MCFJ97		/

PA = *P. aeruginosa*, SA = *S. aureus*, CA = *C. albicans*, AF = *A. fimigatus*, NF =normal nasal flora,

Table S2 Prophage primers used in this study

**Primer name**	**Sequence (5' →3')**	**Gen region targeted**	**Amplicon length (bp)**	**Reference**
PfUa-F	GTGTCGATCAAGATCCACCA	Zonula occludens toxin (*zot*)	872	(1)
PfUa-R	GGAGGAAGAAAGCTATTCGCA			
PfUb-F	TTGTGTACGACAGCGGGAA	Prophage replication module containing an IS element	569-593 or 1242	(2)
PfUb-R	TCAATTCGCCTTTTTCGGC			
Pf1-F	GTGCTTGTTGCCTTCGTTATTC	Capsid protein (*coaA*)	519	
Pf1-R	GTGGAGCCTTTGACCAGATAG			
Pf4-F	TCGAATTCCGCTTCCATCAC	Intergrase gene (*int*)	1001	(1)
Pf4-R	CCTGATGCTTGGTCAGGTACG			
Pf5-F	CGGGAATCGTATTGAGCCGA	Capsid protein (*coaA*)	440	(2)
Pf5-R	GAGGACAGCCAGGGTCATTC			

Table S3 Significant pairwise differences (p-values for origin vs. day *n* in minimal inhibitory concentrations (MICs) during biofilm growth

Antibiotic	Day 1	Day 2	Day 3	Day 4	Day 5
**CF-75**					
**PIP/TAZ**	0.0169	<0.0001	ns	ns	ns
**CAZ**	<0.0001	<0.0001	<0.0001	<0.0001	<0.0001
**FEP**	<0.0001	<0.0001	<0.0001	<0.0001	<0.0001
**ATC**	<0.0001	<0.0001	<0.0001	<0.0001	<0.0001
**MER**	ns	ns	ns	ns	ns
**IMI**	ns	ns	ns	ns	ns
**CIP**	<0.0001	<0.0001	<0.0001	<0.0001	<0.0001
**LEV**	<0.0001	ns	ns	ns	ns
**AMI**	<0.0001	<0.0001	<0.0001	<0.0003	<0.0001
**TOB**	<0.0001	<0.0001	<0.0001	<0.0001	<0.0001
**COL**	<0.0001	<0.0001	<0.0001	<0.0001	<0.0001
**ATM**	<0.0001	<0.0001	<0.0001	<0.0003	<0.0001
**PAO1**					
**PIP/TAZ**	ns	ns	ns	ns	ns
**CAZ**	ns	ns	ns	ns	ns
**FEP**	ns	ns	ns	ns	ns
**ATC**	ns	ns	ns	ns	ns
**MER**	ns	ns	ns	ns	ns
**IMI**	ns	ns	ns	ns	ns
**CIP**	ns	ns	ns	ns	ns
**LEV**	ns	ns	0.003	ns	ns
**AMI**	<0.0001	<0.0001	<0.0001	<0.0003	<0.0001
**TOB**	ns	ns	ns	ns	ns
**COL**	ns	ns	ns	ns	ns
**ATM**	ns	ns	ns	ns	ns

Statistical analysis was performed using two-way ANOVA followed by Tukey's multiple-comparison test (GraphPad Prism 9).Only *p*-values < 0.05 are shown.

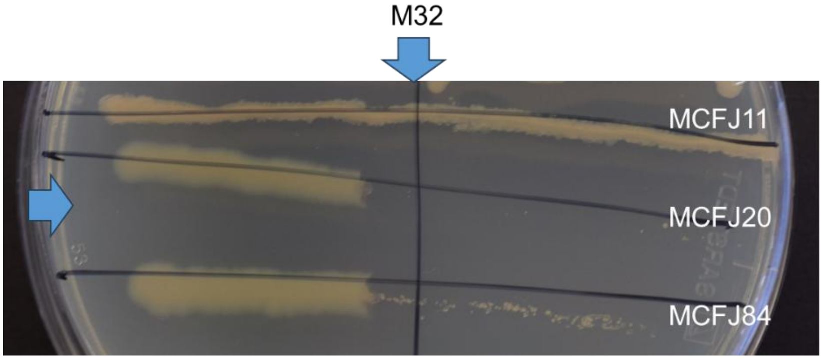

Figure S1 Exemplary test of cross-streak agar assay. The phage (here M32) was inoculated vertically and the P. aeruginosa strains (here MCFJ11, MCJF20, and MCJ84) were inoculated horizontally from left to right and plate was incubated over night at 37°C. Confluent growth after the phage inoculum was interpreted as resistance (MCFJ11), absent growth as susceptible (MCFJ20), and single colonies after the phage inoculum as reduced susceptibility (MCFJ84)

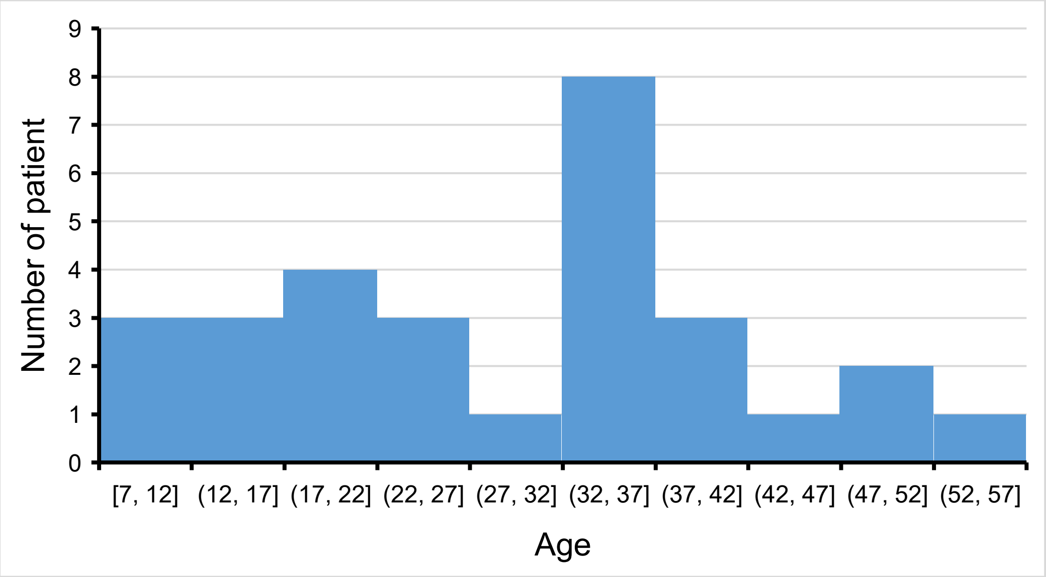

Figure S2 Range of the patients' age included in this study.

	Mucoid	CAZ	PIPI/TAZ	MER	CIP	TOB	COL	AMI	PfUa	PfUb	Pf1	Pf4	Pf5	NP1	NP3	M32
Age	0.03	- 0.04	- 0.05	- 0.08	- 0.08	0.08	- 0.23	0.01	0.05	- 0.02	0.29	- 0.13	0.08	0.08	- 0.08	- 0.03
Mucoid		- 0.04	0.03	0.13	0.17	- 0.19	- 0.23	- 0.11	- 0.13	0.00	0.03	0.03	0.02	0.17	0.07	0.37
CAZ			0.72	0.50	0.30	0.24	0.12	0.46	- 0.06	0.03	- 0.07	0.07	0.11	- 0.27	0.17	- 0.23
PIPI/TAZ				0.52	0.34	0.30	0.02	0.35	0.02	0.10	0.02	0.07	0.10	- 0.26	0.02	- 0.22
MER					0.53	0.09	0.11	0.16	- 0.27	- 0.27	- 0.28	- 0.15	0.16	- 0.25	- 0.11	- 0.11
CIP						0.17	0.00	0.06	- 0.18	- 0.23	- 0.18	0.12	0.09	- 0.08	- 0.08	0.14
TOB							0.22	0.60	0.21	- 0.06	- 0.02	- 0.04	- 0.11	0.04	- 0.01	0.02
COL								0.26	- 0.15	- 0.11	- 0.26	- 0.22	- 0.14	- 0.03	0.01	0.13
AMI									- 0.01	- 0.11	0.03	- 0.19	0.06	- 0.10	0.16	- 0.13
PfUa										0.71	0.35	0.44	0.08	0.19	0.32	0.13
PfUb											0.29	0.56	0.05	0.22	0.36	0.17
Pf1												0.11	- 0.16	- 0.01	0.32	0.05
Pf4													0.11	0.24	0.01	0.26
Pf5														- 0.17	- 0.18	- 0.15
NP1															- 0.01	0.68
NP3																0.09

Scale
- 1.00
- 0.50
- 0.25
0.25
0.50
1.00

Figure S3 Pearson correlation matrix of the relevant isolates' properties. The values for the calculation were used as follows: Mucoidity absent = 0, present = 1; antibiotic susceptibility = 0, intermediate MIC = 1, and resistance = 2; prophage signature absent = 0, present = 1; phage susceptibility = 0, reduced phage susceptibility = 1, phage resistance = 2. The Pearsons rank (r_p) is shown, and the colour scheme (see scale) indicates the positive (green) and negative (correlation). For levofloxacin and aztreonam no r_p could be calculated as there were to less MICs co-assessed for both antibiotics in the isolates. Antibiotics abbreviation see Fig. S2.

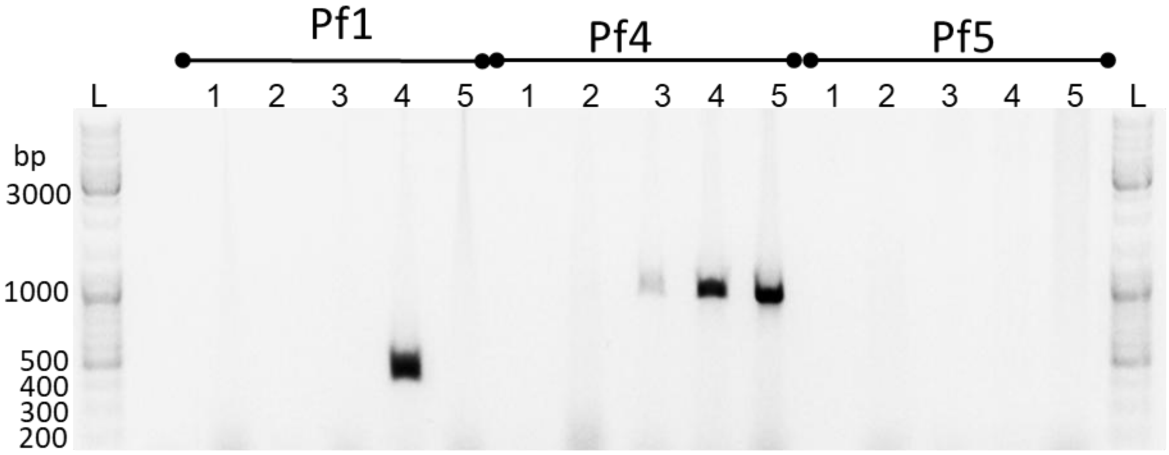

Figure S4 Phage detection in the supernatants of biofilms of selected strains. Reactivated phages were specified by PCR after day 5 of biofilm growth by the Pf1, Pf4 and Pf5 specific primers in CF-PA06 (No. 2), CF-PA75 (No.3), CF-PA83 (No. 4) PAO1 (No. 5), No. 1 = negative control, L = molecular ladder.

Additional results

Demographics of the CF cohort

A total of 51 PA isolates derived from 49 specimens of 29 CF patients were examined retrospectively in this study. From 12 of these patients, multiple isolates were obtained and examined: In six cases, two phenotypically different *P. aeruginosa* isolates were derived from a single sample, and in two cases, three different isolates were identified from one sample. The remaining isolates from these patients were collected during different visits. Only one isolate was analysed for the other 17 patients, even though *P. aeruginosa* was isolated from them at other visits. Due to the small sample size and the number of repeated isolates from the same patients, we do not consider a demographic analysis of the patients useful for the project's purpose. However, factors such as age and gender could play a role, which is why these were recorded descriptively for each isolate. The range of the patients' age ranged from 7 to 57 years, with some more frequent samples at age range between 32 and 35 and an average age of 30 (Supplementary Material Figure S2). The sex distribution was almost equal, with 45% male and 55% female patients. The specimen distribution was 41% sputum (n = 20), 27% throat swab (n = 13) and 14% nasal lavage (n = 7). Mucoid growth on agar plates was observed in 49% (n = 24) of the isolates.

Correlated to the number of specimen (n = 41), other species were found in 61.0% the samples, with *S. aureus* being most prominent (29.3%), followed by unidentified oral flora(24.4%). *Escherichia coli*, *Candida albicans* and *Aspergillus fumigatus* were found only once and in each case were present alongside the *P. aeruginosa* isolates used in this study.

Resistances of the *P. aeruginosa* strains

For an initial detection of *P. aeruginosa* with pulmonary exacerbation, intravenous (iv) antibiotic therapy is recommended. Various substances can be used, typically depending on the resistance profile of the isolates. To improve the success of eradication, therapy is usually followed by inhalation of tobramycin or colistin, along with oral administration of ciprofloxacin (15).

Resistance profiles of the 51 isolates were determined during routine diagnostics as minimal inhibitory concentration (MIC) values and interpreted according to the EUCAST guideline as susceptible, intermediate, or resistant (Supplementary Material Figure S3A). All isolates were tested against ceftazidime (CAZ), piperacillin/tazobactam (PIP/TAZ), meropenem (MER), ciprofloxacin (CIP), tobramycin (TOB; for one patient TOB was not determined for an unknown reason), colistin (COL), and amikacin (AMI). These antibiotics constitute the essential anti-pseudomonal therapy in Germany (15).

Susceptibility was low for CAZ (2% susceptible, 65% intermediate, 33% resistant) and PIP/TAZ (4% susceptible, 61% intermediate, 35% resistant). MER showed 53% susceptibility, 33% intermediate, and 14% resistance. CIP resistance was high at 65%, with 10% susceptible and 25% intermediate. TOB susceptibility was 57% and resistance 43%, with no intermediate results. COL exhibited the highest susceptibility rate (82% susceptible, 18% resistant). AMI showed 61% susceptibility and 39% resistance.

Antimicrobial susceptibility patterns showed generally positive correlations between compounds (Supplementary Material Figure S3), consistent with the frequent occurrence of multidrug resistance in CF isolates (16).

References

1. Mooij MJ, Drenkard E, Llamas MA, Vandenbroucke-Grauls CMJE, Savelkoul PHM, Ausubel FM, et al. Characterization of the integrated filamentous phage Pf5 and its

involvement in small-colony formation. Microbiology (Reading) [Internet]. 2007 Jun [cited 2023 Oct 10];153(Pt 6):1790–8.

2. Knezevic P, Voet M, Lavigne R. Prevalence of Pf1-like (pro)phage genetic elements among Pseudomonas aeruginosa isolates. Virology. 2015 Sep 1;483:64–71.
